# News and Notes

**Published:** 1911-09

**Authors:** 


### NEWS and NOTES

### PROCEEDINGS OF THE AMERICAN PROCTOLOGIC SOCIETY AT LOS ANGELES, CAL., JUNE 26 and 27, 1911.

### EXTRACTS FROM THE REPORT OF PROCTOLOGIC LITERATURE FROM MARCH, 1910 TO MARCH, 1911.

By Samuel T. Earle, M. D., of Baltimore, Md.

In Samuel T. Earle's review of Proctologic Literature from March, 1910, to March, 1911, he quotes from the following authors, giving the salient points from each of their papers:

Harrison Cripps, British Medical Journal, Vol. 1, 1910, p. 292, endorsing Mummery's criticism of Whitehead's operation for hemorrhoids.

Dr. F. C. Wallis, British Medical Journal, Vol. 1, 1910, p. 415, in defence of Whitehead's operation.

Dr. Donald C. Balfour, Rochester, Minn., Annals of Surgery, Vol. II, 1910, p. 239, gives Dr. W. J. Mayo's method of Anastomosis between the sigmoid and rectum.

Dr. Charles H. Peck, New York City, Annals of Surgery, 1910, Vol. LI, p. 242, describes a method of excising the rectum for cancer by the perineal route.

Dr. Norman Porritt, London Lancet, 1910, Vol. 1, page 360, describes a simple and efficient operation for hemorrhoids.

Dr. L. P. Lockhart Mummery, London Lancet 1910, Vol. I, p. 641, describes a new operation for prolapse of the rectum.

Mr. Heaton C. Howard, London Lancet 1910, Vol. 1, p. 240, showed a case of stricture of the rectum treated by injection of Fibrolysin, which were given three times a week.

Dr. Walton Martin, Annals of Surgery 1910, Vol. LI, p. 125, reported a case of anastomosis between the sigmoid and rectum by invagination.

Dr. Joseph A. Blake, Annals of Surgery 1910, Vol. LI, p. 261, gives an unusual method of anastomosis in cases of carcinoma of the rectum.

Extracts from a statistical report of 120 cases of removal of the rectum for cancer by Dr. William J. Mayo, Annals of Surgery 1910, Vol. LI, p. 895.

Dr. W. Sampson Handley, in his second Hunterian Lecture, gives some very interesting and instructive suggestions about the extension of cancer of the rectum by the lymphatic system, British Medical Journal 1910, Vol. I, p. 927.

A series of instructive papers on excision of the rectum for carcinoma can be found in the British Medical Journal 1910, Vol. I, by the following writers:

Charles A. Morton, page 1378, Harrison Cripps, page 1323, F. Swinford Edwards, page 967, W. Bruce Clarke, page 1023, P. Lockhart Mummery, page 1144, W. Ernest Miles, page 1203.

James Swain, British Medical Journal 1910, Vol. 1, p. 361, advocates strongly the removal of all lymph glands in cancer of the rectum.

Dr. E. K. Scott, Boise, Idaho, Northwest Medicine, Vol. II, No. 3, p. 85, a plea for more thorough examinations of the rectum for carcinoma by the general practitioner.

Dr. C. L. Gibson, Annals of Surgery, Vol. LI, p. 116, gives a special method for end-to-end Intestinal Anastomosis by the Invagination Method, in cases where other methods would be impracticable. Sigmoid Replaced by Small Intestine. Reichel, Verhand d. Deutsch. Gesell. f. Chir., April, 1910.

Dr. Wilson, Annals of Surgery, February, 1911, p. 223, speaks of the association of diverticuli and carcinoma in the lower bowel.

DeWitt Stetten, Festschrift of the German Hospital, New York, 1909, published two most interesting observations on the co-existence of tuberculous ulcers and carcinoma of the large intestine.

Dr. Wyllys Andrews, Surgery, Gynecology and Obstetrics, January, 1911, p. 63, gives an interesting account of a new form of industrial Accident-Pneumatic Rupture of the Intestine.

Baird, Semaine Medicale, November 30, 1910, Vol. XXX, No. 48, p. 565, recounts a case showing this rather unusual type of Hirschsprung's disease—Idiopathic Dilatation of the Rectum.

Treatment of Painful Fissures and Piles by High Frequency Currents.—A Teirlinck, Gand, Belgium. The Proctologist, December, 1910.

---

The following article by Dr. A. Teirlinck, of Gand, Belgium, was read by title:

*How Can an Infected Sigmoid Diverticulum be the Cause of a Rethro-Peritoneal Abscess.*

In the present state of abdominal surgery, the appendix is frequently regarded as the chief cause of all abdominal troubles.

Recently numerous works have been published concerning sigmoiditis and perisigmoiditis. Diverticular abscesses are not as frequent as appendicular abscesses. It should be borne in mind that the sigmoid is often located in the right iliac fossa and diverticular abscesses may be mistaken for appendicular trouble.

In the young, the sigmoid flexure is free and communicates with the retro-mesenteric and pre-aortic cellular tissues by the tissue of the meso-colon. Infection can be transmitted from the diverticula into the retro-peritoneal cellular tissue by three means —the connective tissue, the lymphatic system, and the venous blood-vessels.

In adults, the sigmoid is adherent to the posterior abdominal wall, and in such cases there is another source of infection,—an external one, due to the numerous anastomoses between the meso-colic glands and the parietal lymphatic system and between the sigmoid blood-supply and that of the retro-peritoneal region.

---

*Some Observations upon the Surgical Anatomy and Mechanism of the Colon.—By Granville S. Hanes, M. D., of Louisville, Ky.*

Until comparatively recent years diseases of the colon and sigmoid, and the surgical anatomy of each, received but scant attention. Recently, however, much valuable information upon this subject has been developed. Robert Coleman Kemp in his work on Diseases of the Stomach and Intestines, says that Dr. J. M. Mathews was the first to call attention to sigmoiditis and diverticulitis of the sigmoid.

The entire length of the large bowel in situ is found to be much shorter than when it is dissected from its attachments. An ordinary thirty-inch colon tube has sufficient length to extend around the lumen of the large bowel to the cecum. While this has not been done in the living individual, it has been done in the cadaver, and radiographs of the same are on record.

It is almost universally believed that ordinary flexible colon tubes can be manipulated in such a way as to traverse the entire course of the large bowel around to the cecum. It has been proven by a number of investigators that such an achievement is impossible in the normal bowel. The average length of the sigmoid is about eighteen inches, and this being a floating portion of the large gut it is almost impossible for an instrument to pass beyond the middle half of the sigmoid. Should such be possible, and the tube enter the descending colon, it would be a physical impossibility for it to pass either the acute angle at the splenic flexure or the hepatic flexure. The failure of instruments to pass high into the bowel has been demonstrated by X-ray pictures.

Dr. Hanes demonstrated the difficulty in passing any instrument through the hepatic and splenic flexures by introducing a thirty-inch No. 20, French, soft rubber catheter into the caput coli in an old appendicostomy case. He failed by any kind of manipulation to pass the catheter through these flexures. The tube was allowed to remain in the head of the colon for twenty-four hours with he hope that peristalsis would carry it around, but this failed. After manipulating the second time, three hours later four inches of the catheter appeared through the anal opening.

He forced bismuth solution into the head of the colon till the wall of the gut was thoroughly distended and then Dr. E. Bruce made a skiograph. No regurgitation into the ileum occurred. This experiment was repeated a number of times with the results as above given. If the ileo-cecal valve allows no reflow into the ileum, then exceedingly large amounts of water injected into the bowel are retained in the large gut, and not a part of the amount passed into the small bowel, as is supposed by some.

In an old appendicostomy case, with the patient on the left side, coal-oil was poured into the colon tube that had been introduced three inches into the rectum. In six and a half minutes the oil was flowing out of the appendicostomy opening. The amount employed was thirty ounces. This clearly demonstrates that liquids will easily pass around the entire colon without flowing through a tube. The point is also made that coal-oil is much less irritating to the mucosa than plain water or ordinary aqueous solutions.

The capacity of the large bowel in situ was measured by temporarily closing the opening of an appendicostomy case and allowing coil-oil to flow into the rectum as long as the patient could tolerate it. At a later date the same experiment was made by allowing oil to flow into the head of the colon. About the same amount of oil was received in each case. After making the same experiments in other cases it was decided that the average large bowel had a capacity varying between fifty and sixty-four ounces.

The capacity of the rectum was ascertained by inverting the patient and placing a colpeurynter at the junction of the sigmoid and rectum, just within the sigmoid. The colpeurynter was then distended with air until no fliud could pass into the sigmoid. Coal-oil was allowed to flow into the rectum until no more could be received. It was then drawn off with a catheter and the average amount was found to be between fourteen and seventeen ounces.

He insists that the Inverted Position (Hanes) is much to be preferred by both patient and operator when any kind of illuminating instruments are to be employed in the rectum or sigmoid.

---

*Have We An Ideal Operation for Internal Hemorrhoids?—A New Hemorrhoidal Clamp.—By A. B. Cooke, M. D., of Nashville, Tenn.*

An ideal operation for internal hemorrhoids must embody the five following surgical principles and precepts:

1. Complete hemostasis.
2. Immediate closure of the operative wounds.
3. Preservation of the function of the parts.

4. Permanency of cure.

5. Due consideration of the factors of safety, simplicity of technic, time required for recovery, and the amount of post-operative discomfort.

The ligature operation violates principle 2.

The clamp and cautery operation falls short with reference to the fourth class of principles in each of its several points.

The Whitehead operation violates principles 1, 3 and 5, and is, moreover, an unnecessary and unjustifiable procedure.

The operation by means of Earl's clamp is a modification of the Whitehead method and a vast improvement upon it, but is apt, likewise, to violate principle 3.

Pennington's enucleation operation is open to criticism under classes 1 and 5 of the surgical principles. In spite of its ingeniousness, it is dangerous.

The Clamp and Suture operation described by the author fulfills all conditions and is entitled to be considered the most nearly ideal of any yet devised.

A new hemorrhoidal clamp designed to facilitate the last named operation was presented and strongly recommended.

---

A Symposium on Constipation embracing seven different parts was presented as follows:

*Etiology of Constipation.—By Horace Heath, M. D., of Denver, Col.*

Dr. Heath mentioned two groups—miscellaneous and mechanical. Under miscellaneous, the author regarded heredity as unimportant, but attention was called to the faulty instruction of children in certain families. He stated that the constipation of infancy was due to undeveloped muscles; and of old age, to inactivity and atonicity.

Under mechanical causes he considered—diet, sedentary life, abnormal positions, angulations, coloptosis, and hypertrophy of the rectal valves.

The predisposing diseases mentioned were colitis, stricture, proctitis, fissure, hemorrhoids, fistula, polypi, enlarged prostate, and malignant growths.

*Physiology of Constipation.—By Samuel T. Earle, of Baltimore, Md.*

In reviewing the Physiology of Constipation in the symposium read before the American Proctologic Society, June, 1911, Earle calls attention to the sensibility of the alimentary canal in connection with its bearing on constipation. It has been shown that the stomach and intestines are quite insensitive to tactile and thermal stimuli, but that the esophagus and anal canal are sensitive. The whole of the alimentary canal is, however, sensitive to distension, which produces at first discomfort, and subsequently pain. The rectum appears to be more sensitive than the rest of the intestines to distension, so that a large fecal mass produces more discomfort when lodged in the rectum than in any other situation. As a result of this, the normal accumulation of feces in the pelvic colon is unaccompanied by any discomfort, whereas, the entry of feces into the rectum at once produces a sensation, which acts as a warning that defecation is necessary. The discomfort produced by the presence of a large mass of feces in the rectum is partly due to the pressure it exerts on the upper extremity of the sensitive anal canal. Prolonged retention of feces in the rectum leads to a blunting of its sensibility, so that comparatively little local discomfort is present in most cases of confirmed constipation. But in acute cases or cases of recent origin, in which the rectum is distended with feces, much discomfort and occasionally severe pain is experienced. On the other hand, even a very large accumulation in the pelvic colon produces little or no discomfort in the intestine itself.

A large fecal accumulation in the rectum presses directly upon the anterior primary divisions of the third, fourth and fifth sacral nerve routes, as they emerge from the sacral foramina. It may, therefore, lead to neuralgic pain referred to the sacrococcygeal region. It is liable to cause suffering more from its constant presence than its severity; it is often as severe when the patient lies down as when he takes exercise, but some relief follows flexion of the lumbar spine. The muscles of the buttocks and back of the thigh, which receive a small part of their sensory and motor supply from the third sacral nerve route, may be the seat of similar pain. Neuralgic pain or parasthesia, in the form of tingling or a sensation of heat or cold may occur in the course of the sciatic nerve, in the back of the thigh, and occasionally the sensation of cramp in the calf is produced. Pain is also occasionally felt in the hip joint, it receives part of its nerve supply from the third sacral nerve. The roots which supply the muscles of the front of the thigh, are situated out of reach of the distended rectum, so that in the exceptional cases in which pain is produced by constipation in this situation, it must be due to pressure exerted by a fecal mass in the iliac colon on the anterior crural nerve; and is accordingly only observed on the left side.

That these neuralgic pains are probably due to the direct presence of a large and hard mass of feces, on the sacral nerve routes is shown by their instantaneous disappearance on completely evacuating the rectum by anemata, a form of treatment which was already advocated by Columnius of Naples at the end of the eighteenth century.

Possibly the erections and seminal emissions, and the frequency of micturition and nocturnal incontinence, which occasionally result from large fecal accumulations in the rectum, are due to direct irritation of the third and fourth sacral nerves, and are not reflex in nature. The spasm of the spinchter ani and levator ani muscles, which has already been described as an occasional complication of the fecal impaction in the rectum, which occurs in constipation, may perhaps in part be due to pressure on the fourth sacral nerve routes.

Neuralgia of the testicles in men and dysmenorrhea in women are sometimes increased by the direct pressure in the rectum on the nervous supply of the testicles and uterus respectively.—Arthur F. Hertz, on Constipation.

*(To Be Continued.)*

